# Human stem cells from single blastomeres reveal pathways of embryonic or trophoblast fate specification

**DOI:** 10.1242/dev.122846

**Published:** 2015-12-01

**Authors:** Tamara Zdravkovic, Kristopher L. Nazor, Nicholas Larocque, Matthew Gormley, Matthew Donne, Nathan Hunkapillar, Gnanaratnam Giritharan, Harold S. Bernstein, Grace Wei, Matthias Hebrok, Xianmin Zeng, Olga Genbacev, Aras Mattis, Michael T. McMaster, Ana Krtolica, Diana Valbuena, Carlos Simón, Louise C. Laurent, Jeanne F. Loring, Susan J. Fisher

**Affiliations:** 1 Center for Reproductive Sciences, University of California San Francisco, San Francisco, CA 94143, USA; 2 Division of Maternal Fetal Medicine, University of California San Francisco, San Francisco, CA 94143, USA; 3 Department of Obstetrics, Gynecology, and Reproductive Sciences, University of California San Francisco, San Francisco, CA 94143, USA; 4 The Eli & Edythe Broad Center for Regeneration Medicine and Stem Cell Research, University of California San Francisco, San Francisco, CA 94143, USA; 5 Human Embryonic Stem Cell Program, University of California San Francisco, San Francisco, CA 94143, USA; 6 Center for Regenerative Medicine, Department of Chemical Physiology, The Scripps Research Institute, La Jolla, CA 92037, USA; 7 Department of Anatomy, University of California San Francisco, San Francisco, CA 94143, USA; 8 StemLifeLine, San Carlos, CA 94070, USA; 9 Department of Pediatrics, University of California San Francisco, San Francisco, CA 94143, USA; 10 Diabetes Center, Department of Medicine, University of California San Francisco, San Francisco, CA 94143, USA; 11 Buck Institute for Research on Aging, Novato, CA 94945, USA; 12 Department of Pathology, University of California San Francisco, San Francisco, CA 94143, USA; 13 Department of Cell and Tissue Biology, University of California San Francisco, San Francisco, CA 94143, USA; 14 Fundación Instituto Valenciano de Infertilidad (IVI), Parc Científic Universitat de València, 46980, Valencia, Spain; 15 Department of Reproductive Medicine, University of California San Diego, La Jolla, CA 92093, USA

**Keywords:** Human embryo, Blastomere, Human embryonic stem cell, Human trophoblast stem cell, Fate specification, Transcriptome, Epigenome

## Abstract

Mechanisms of initial cell fate decisions differ among species. To gain insights into lineage allocation in humans, we derived ten human embryonic stem cell lines (designated UCSFB1-10) from single blastomeres of four 8-cell embryos and one 12-cell embryo from a single couple. Compared with numerous conventional lines from blastocysts, they had unique gene expression and DNA methylation patterns that were, in part, indicative of trophoblast competence. At a transcriptional level, UCSFB lines from different embryos were often more closely related than those from the same embryo. As predicted by the transcriptomic data, immunolocalization of EOMES, T brachyury, GDF15 and active β-catenin revealed differential expression among blastomeres of 8- to 10-cell human embryos. The UCSFB lines formed derivatives of the three germ layers and CDX2-positive progeny, from which we derived the first human trophoblast stem cell line. Our data suggest heterogeneity among early-stage blastomeres and that the UCSFB lines have unique properties, indicative of a more immature state than conventional lines.

## Abstract (precis)

**Highlighted article:** Human ESCs derived from individual blastomeres of 8- and 12-cell human embryos have unique properties, provide insights into early human embryogenesis and enable human trophoblast stem cell derivation.

## INTRODUCTION

For many reasons, relatively little is known about human preimplantation development. The small number of cells makes embryos of any species difficult to study. In humans, the technical difficulties are compounded by other challenges. Genetic variation among individuals could contribute to developmental differences, a well-appreciated phenomenon in the mouse (Dackor et al., 2009), which is difficult to assess in humans owing to the limited availability of embryos that are donated for research. In mice, culturing embryos leads to changes in gene expression patterns, such as a reduction in the differences between the transcriptomes of the inner cell mass (ICM) and the trophectoderm (Giritharan et al., 2012). In some countries, government regulations preclude the use of federal funds for research on human embryos and their derivatives, or prohibit this work altogether. Despite these inherent difficulties, there are compelling reasons for studying the molecular underpinnings of human embryogenesis. Assisted reproductive technologies have as their cornerstone *in vitro* fertilization (IVF) and the subsequent growth of embryos. However, the culture methods are largely based on conditions optimized for mouse embryos (Quinn, 2012). Likewise, despite decades of searching for biomarkers, selection of embryos for transfer is largely based on morphological criteria (Gardner and Schoolcraft, 1999). Beyond assisted reproductive technologies, methods for generating cells that will be deployed in human embryonic stem cell (hESC)-based therapies will benefit from an understanding of the pathways that govern their genesis.

Human preimplantation development is charted according to several crucial milestones, which are discernable at the light microscopic level. At day 3 postfertilization, the embryo is a solid ball of morphologically similar cells. By day 5, at the early blastocyst stage, segregation of the embryonic and extra-embryonic lineages is first apparent. The trophoblast (TB) cells that form the outer surface of the embryo mediate attachment to the uterine wall and contribute to the placenta. The inner cell mass (ICM) is clustered at one pole of the interior. Prior to the late blastocyst stage, the ICM is partitioned into the flattened hypoblast, the future extra-embryonic endoderm, which is in direct contact with the fluid-filled blastocyst cavity. The epiblast, the source of embryonic precursors, occupies the space between the hypoblast and the TB.

Most of what we know about human preimplantation development, in mechanistic terms, has been inferred from the analogous stages in model organisms. For example, investigators have immunolocalized POU5F1 (POU domain class 5 transcription factor 1; also known as OCT4) and CDX2 (caudal type homeobox 2) in human embryos because gene deletion studies in mice show that these transcription factors are required for formation of the intra- and extra-embryonic lineages, respectively (Nichols et al., 1998; Strumpf et al., 2005). In this species, Cdx2 binds to Tcfap2 (Tfap2e – Mouse Genome Informatics) sites in the *Pou5f1* promoter, shutting off transcription. Notably, the promoters of the bovine and human *POU5F1* genes lack these binding sites, suggesting mechanistic differences among species in the first lineage decision, and predicting the divergence of other downstream programs (Berg et al., 2011). In support of this concept, the expression patterns of POU5F1 and CDX2 follow different kinetics in mouse and human embryos with transient co-expression of both factors in some cells (Niakan and Eggan, 2012). Moreover, less than 5% of POU5F1, NANOG and CTCF sites are homologously occupied in human and mouse embryonic stem cells (Kunarso et al., 2010). Researchers are also using global strategies to profile transcriptional activation and gene expression during human embryonic development (Zhang et al., 2009; Fang et al., 2010; Vassena et al., 2011; Altmäe et al., 2012). These data enable *in silico* assembly of pathways that guide crucial developmental transitions. Yet we still lack insights into fundamental aspects of human embryonic and extra-embryonic development, including when and how fate specification occurs. Approaches for directly addressing these questions are limited. hESCs, which are derived from human embryos, and induced pluripotent stem cells (iPSCs) are currently the best models for functional analyses of early developmental processes in our species.

Accordingly, our group has been interested in deriving hESCs from embryos at earlier stages than the blastocysts that are commonly used for this purpose. Previously, in collaborative studies, we reported the derivation of hESC lines from individual blastomeres of early-stage human embryos that went on to form blastocysts (Chung et al., 2008). We reasoned that the opposite approach, deriving multiple lines from single cells of individual early-stage human embryos, could give us important insights into the properties of these cells. Here, we report the results of experiments that tested this hypothesis.

## RESULTS

### hESC derivation from single related blastomeres

This study was designed to determine whether hESCs derived from early-stage embryos had unique properties compared with conventional lines that are typically derived from later-stage blastocysts. As a first step, we established hESC lines from individual blastomeres of five embryos, four at the 8-cell stage and one at the 12-cell stage. One couple donated all the embryos. We removed single cells from each embryo and cultured them in individual drops of medium on human foreskin fibroblast (HFF) feeders in a physiological oxygen environment of 8% O_2_ according to published methods (Chung et al., 2008; Ilic et al., 2009). Four blastomeres from one embryo, three from another, and single blastomeres from the remaining embryos formed lines, designated UCSFB1-10 (Fig. S1A). We attributed the derivation of different numbers of lines from different embryos to the technical difficulties involved in blastomere biopsy. Removing single cells from cleavage-stage human embryos requires the application of forces strong enough to remove individual cells, which are tightly adhered to one another.

We used standard methods to characterize the UCSFB hESC lines. Karyotyping showed that 9/10 lines were euploid. One was 46 XX and 8 were 46 XY. The line that was derived from the 12-cell stage embryo (UCSFB10) was tetraploid (92 XXYY) and maintained this chromosome complement through early passages. Aneuploidy of this line could reflect frequently observed genetic mosaicism among the cells of human pre-implantation embryos, which often resolves (Barbash-Hazan et al., 2009). In the mouse, tetraploid cells segregate to the yolk sac endoderm and TB lineages, whereas diploid cells give rise to the embryo (Nagy et al., 1990). Although we have no data in this regard, we are interested in their relative potential to form extra-embryonic versus embryonic descendants.

Immunolocalization showed that UCSFB1-9 expressed antigens that are associated with pluripotency: POU5F1, NANOG, TRA-1-60 and SSEA-4 (Fig. S1B). The nine euploid lines formed embryoid bodies (EBs) that underwent specification of the three germ layers as shown by expression of markers of ectodermal (β-III-tubulin), mesodermal (smooth muscle actin) and endodermal (α-fetoprotein) derivatives (Fig. S1C). When transplanted in Matrigel plugs under the skin of nude mice, they also formed teratomas that contained cell types representing the three germ lineages (e.g. neuroectoderm, intestinal epithelium, chondrocytes/cartilage and melanocytes; Fig. S1D). Thus, as determined by standard measures that are applied to conventional hESCs and iPSCs, UCSFB1-9 were pluripotent.

**Fig. 1.**
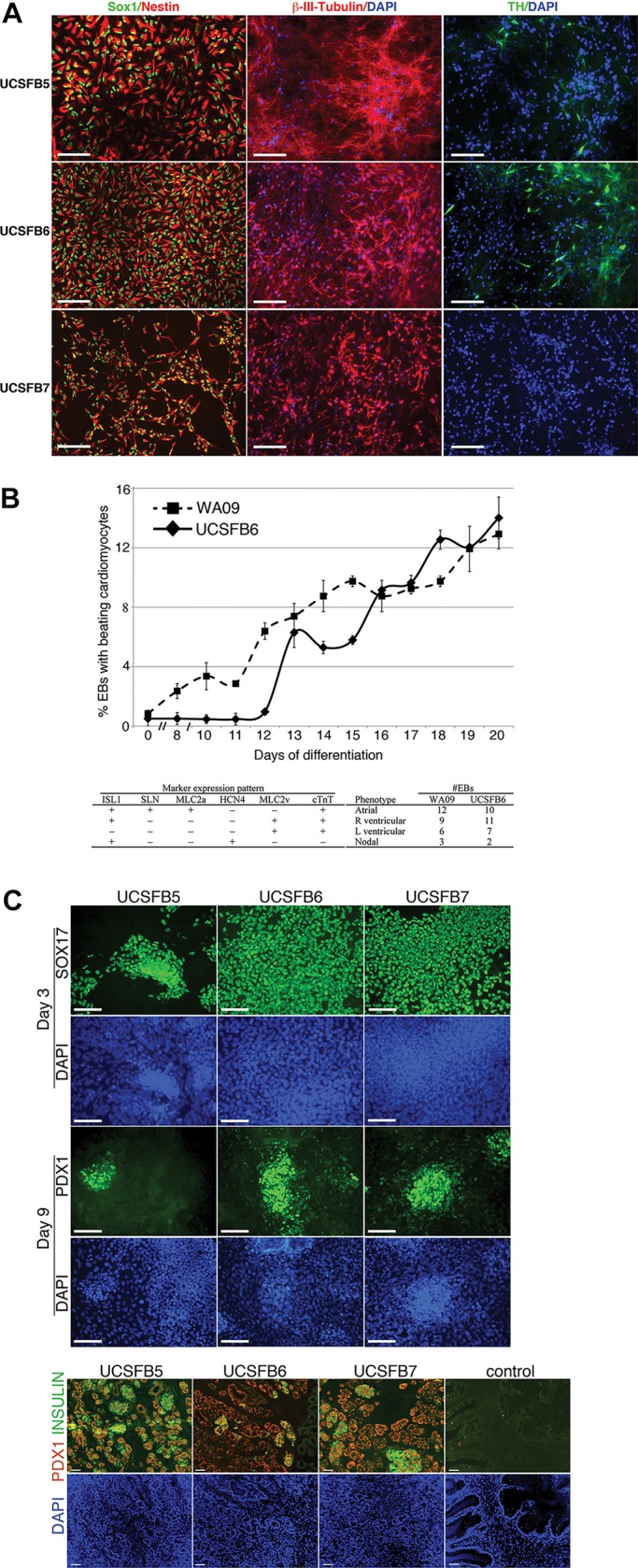
**Directed differentiation of the UCSFB lines into neuronal precursors, cardiomyocytes, endocrine precursors and pancreatic endoderm.** (A) Neuronal precursors derived from UCSFB5-7 expressed SOX1 and nestin (left panels) and β-III-tubulin (middle panels). Two of the lines tested (UCSFB5 and USCSFB6) formed dopaminergic neurons as assessed by immunostaining for tyrosine hydroxylase (TH, right panels). Scale bars: 100 µm. (B, graph) In duplicate, rotary orbital suspension was used to form 300 EBs of uniform size from single cell suspensions of UCSFB6 and WA09 hESCs. Over 20 days, the percentage of adherent EBs demonstrating spontaneous contractile activity in differentiation medium was scored. UCSFB6 formed cardiomyocytes with the same efficiency as conventional hESCs by day 20 (14.0±1.4% versus 12.9±1.0%; *P*>0.1, Student's *t*-test). (B, table) Distribution of cardiomyocyte subtypes differentiated from UCSFB6 compared with WA09 hESCs. qRT-PCR showed that EBs formed embryonic atrial, right and left ventricular, and specialized conduction (nodal) cells with efficiencies similar to WA09 cells (*N*=30; *P*=0.70 [χ2 1.41, DF 3]). (C, upper panels) UCSFB5-7 formed endocrine precursors and pancreatic endoderm. Immunostaining of UCSFB lines cultured in conditions that promoted endoderm differentiation showed that they expressed SOX17 and PDX1, markers of endocrine precursors. (C, lower panels) When transplanted under the kidney capsule of SCID-beige mice for 100 days, UCSFB5-7 immunostained for Pdx1 and insulin, markers of pancreatic endoderm. Scale bars: 100 µm.

Next, we explored the potential of a subset of the UCSFB lines to form more mature derivatives of the three embryonic germ layers. With regard to ectoderm, UCSFB5-7 generated neuronal precursors as demonstrated by immunostaining for SOX1, nestin and β-III-tubulin (Fig. 1A). However, only B5 and B6 gave rise to dopaminergic neurons as shown by staining for tyrosine hydroxylase. As to mesoderm, UCSFB6 robustly formed EBs with spontaneous contractile activity; qRT-PCR analyses showed that they differentiated into cells that expressed markers characteristic of embryonic atrial, right and left ventricular, and specialized conduction (nodal) cells (Fig. 1B). As to endoderm, UCSFB5-7 robustly formed endocrine precursors and pancreatic endoderm *in vitro* and insulin-producing cells *in vivo* (when transplanted into mice) (Fig. 1C). Thus, based on the results shown in Fig. S1 and Fig. 1, we concluded that the UCSFB cells met the standard definition of an hESC line.

### Blastomere- and blastocyst-derived hESCs differ at the transcriptional level

**Fig. 2.**
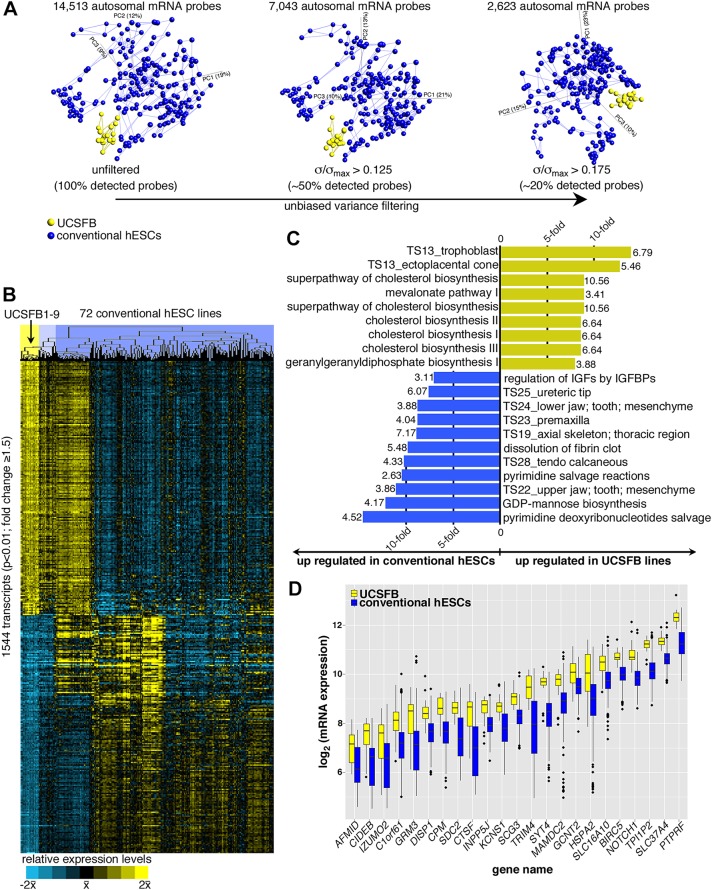
**The UCSFB lines and conventional hESCs had distinct transcriptomes.** (A) A 3D principal component analysis and an unbiased nearest neighbor clustering plot of global transcriptional profiling data (shown in B) showed that the UCSFB lines were distinct from 228 samples of 72 conventional hESCs according to: (left) 100% of the detected autosomal transcripts; (middle) the most variable transcripts at a 50% level; and (right) the most variable transcripts at a 20% level. (B) Heatmap of the gene expression data (*P*<0.01) shown in A. Colors at the top indicate lines as follows: yellow, UCSFB lines; blue, conventional lines; light blue, conventional hESCs that were distinguished from UCSFB by a single bifurcation in the array tree dendrogram (detailed in Fig. S2). (C) Gene enrichments, as determined by GREAT, showed that mRNAs involved in trophoblast and cholesterol biosynthetic pathways were upregulated in the UCSFB lines. Conversely, mRNAs that regulate various aspects of embryonic development were upregulated in the conventional lines. Bars depicting the fold enrichment for each gene set were annotated with the −log10 *P*-values. (D) mRNA expression levels for a gene set in a Naïve Bayes classifier that distinguished UCSFB lines from conventional hESCs. They included regulators of fundamental developmental processes such as *DISP1* and *NOTCH1.* TS, Theiler stage.

To determine if there were transcriptional variations among blastomere and conventionally derived hESCs, we compared the transcriptomes of two biological replicates of UCSFB1-9 to those of 228 samples from 72 conventional hESC lines that were generated on the same microarray platform (Nazor et al., 2012). Initially, we plotted sample relationships in a 3D principal component analysis (PCA) matrix according to all detected autosomal mRNA probes (Fig. 2A, left PCA matrix). In this analysis, Euclidean distance measures were used to connect each sample to its two nearest neighbors, forcing each UCSFB sample to choose at least one nearest neighbor other than its corresponding biological replicate. UCSFB1-9 formed a distinct cluster without nearest neighbor edges drawn between these lines and those derived by conventional means. To test the stability of this clustering, we performed an unsupervised, variance-based reduction to the most variable probes on the array with the threshold set at 50% or 20% and repeated this analysis (Fig. 2A, middle and right PCA plots, respectively). The results showed that the robust separation of UCSFB cells from conventional hESC lines was maintained. Thus, we sought to explore the molecular bases of differences between pluripotent cells derived from blastomeres and blastocysts.

Two major expression patterns emerged from the 1544 mRNAs that were differentially expressed by ≥1.5-fold (*P*<0.01) between the UCSFB (Fig. 2B, yellow shaded portion of the cluster) and conventional hESC lines (Fig. 2B, blue shaded portion of the cluster; Table S3A). First, approximately half of the mRNAs were uniformly highly expressed in the UCSFB lines relative to >75% of the conventional hESCs. Conversely, a roughly equal number of mRNAs were strictly repressed in the UCSFB lines, but highly expressed in a majority of the conventional hESC lines. Functional enrichments were determined for these gene sets using the Genomic Regions Enrichment of Annotations Tool (McLean et al., 2010). The subset of mRNAs with elevated expression in the UCSFB lines was most highly enriched for genes expressed by trophoblasts, the ectoplacental cone (polar trophectoderm of the mouse) or involved in metabolism, particularly cholesterol biosynthesis (Fig. 2C; Table S3B). These enrichments were paralleled by a striking reduction in UCSFB expression of mRNAs encoding components of pyrimidine salvage pathways, glycosylation, insulin-like growth factor family members, fibrin clot dissolution and genes that function during developmental/morphogenic processes (Fig. 2C; Table S3B). Thus, a subset of the pathways that were upregulated in the UCSFB lines functioned in trophoblast and those that were differentially expressed by conventional lines pointed to the initiation of differentiation processes.

Of the conventional lines, 15 were more similar to the UCSFB hESCs, separated by a single bifurcation in the array tree dendrogram (Fig. 2B, light blue shaded portion of the cluster). Nevertheless, 953/1544 transcripts from the overall analysis were significantly differentially expressed (*P*<0.01) between the blastomere-derived cells and this subset of blastocyst-derived lines (Fig. S2A; Table S3C). Of the 498 transcripts that were upregulated in the UCSFB lines, mRNAs associated with trophoblasts and cholesterol biosynthesis pathways continued to be the most significant (Fig. S2B; Table S3D). This analysis also revealed higher expression of mRNAs encoding organelle and plasma membrane components, possibly reflecting the importance of assembling the cellular machinery in cleavage-stage embryos. Fig. S2C is a heatmap of the 50 most significantly upregulated UCSFB transcripts in this smaller comparison. Of the 455 mRNAs that were downregulated in the UCSFB lines, ∼10% encode gene products that are expressed in mitochondria. The others function in signaling pathways (*IL1A*, *TNF*, *TCRB*, *NGFR*, *FAS* and *NTRK1*). Fig. S2D is a heatmap of the 50 most significantly upregulated conventional hESC transcripts in this smaller comparison. Thus, we concluded that the UCSFB lines had significantly different transcriptomes compared with conventional hESCs.

### Gene expression classifier for single blastomere-derived hESCs

Given the robust transcriptional differences between conventional hESCs and the UCSFB lines, we developed an unbiased classifier for the latter cells. For this analysis, we incorporated an additional 88 gene expression profiles of conventional hESCs from another microarray platform (Illumina HT12v4). We used half of these data to train the model and the other half for testing. The Naïve Bayes classifier we developed had an accuracy of 98.3% (sensitivity=91.7%, specificity=97.9%). Fig. 2D shows the mRNA expression patterns of 23 genes that contributed to this model. Several have interesting functions with potentially important roles in early development. For example, NOTCH1 is required for stem cell homeostasis (Gerhardt et al., 2014), SDC2 (syndecan 2) binds growth factors (Leonova and Galzitskaya, 2013) and DISP1 (dispatched homolog 1) mediates long-range hedgehog signaling (Etheridge et al., 2010). Thus, we developed a classifier that could be applied to other pluripotent cells.

### Blastomere- and blastocyst-derived hESCs differ at the methylome level

**Fig. 3.**
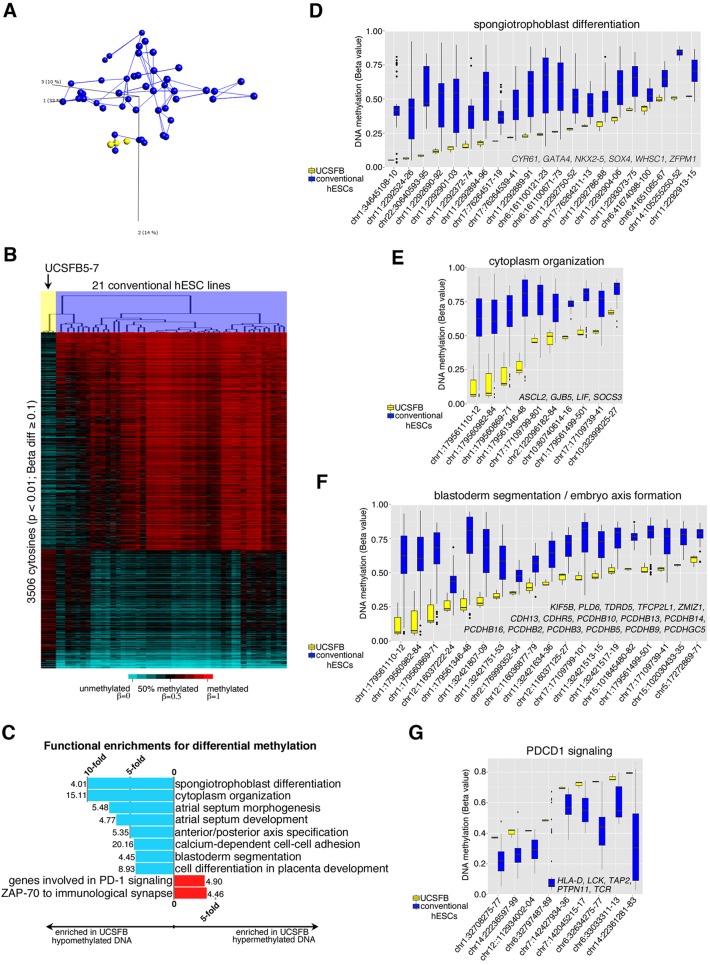
**The UCSFB lines were hypomethylated compared with conventional hESCs in genomic regions that control trophoblast differentiation and basic developmental processes.** (A) A 3D principal component analysis and an unbiased nearest neighbor clustering plot of the methylation data (shown in B) differentiated the UCSFB (yellow) and conventional hESC (blue) lines. (B) Heatmap showing differentially methylated cytosines (*P*<0.01, beta-value≥0.1) of UCSFB5-7, which were derived from a single embryo, compared with 46 samples from 21 conventional hESC lines. (C) Functional enrichments for differentially methylated cytosines between UCSFB lines and conventional hESCs as determined by GREAT. Bars depicting the fold enrichment for each gene set were annotated with the −log10 *P*-values. The hypomethylated regions, which dominated, encoded genes involved in trophoblast/placental differentiation and key aspects of developmental processes such as cytoplasmic organization, cell adhesion and blastoderm segmentation. The hypermethylated regions were involved in PDCD1 signaling and ZAP-70 functions at the immunological synapse. (D-G) Box and whisker plots of genes driving enrichments for spongiotrophoblast differentiation, cytoplasmic organization, blastoderm segmentation/embryo axis formation and PD-1 signaling, respectively. Cytosine genomic coordinates are plotted on the *x*-axis. The associated transcripts are listed in the bottom right-hand corners of the panels.

DNA methylation, a powerful epigenetic mechanism to regulate gene expression, can be indicative of a cell's developmental history and functional maturity (Hemberger and Pedersen, 2010). Using the Illumina Infinium 450 K DNA Methylation BeadChip platform, we investigated whether the observed transcriptional differences among the UCSFB and conventional lines were paralleled by distinct DNA methylation patterns. We profiled UCSFB5-7, which were derived from the same embryo, and compared them to a panel of 46 DNA samples from 21 conventional hESC lines. When sample relationships were plotted in a 3D-PCA matrix according to cytosine methylation, the UCSFB samples clustered at one extreme of the distribution without nearest neighbor edges drawn between these lines and those derived by conventional means (Fig. 3A). *In toto*, we identified 2271 cytosines that were hypomethylated and 1235 that were hypermethylated in the UCSFB lines relative to the conventional hESCs (Fig. 3B; Table S4A). In agreement with the functional enrichments observed for gene expression, the hypomethylated CpGs were in chromosomal locations that are highly enriched for trophoblast/placental genes and early embryonic differentiation processes (Fig. 3C; Table S4B). The functional categories included spongiotrophoblast differentiation (Fig. 3D), cytoplasmic organization (Fig. 3E) and blastoderm segmentation/embryo axis formation (Fig. 3F). The hypermethylated CpGs in the UCSFB lines relative to conventional lines were in gene regions that are involved in peripheral immune tolerance, specifically PD-1 (Fig. 3G) and ZAP70 signaling (data not shown). Repression of these pathways is consistent with the need to block maternal immune rejection of the hemi-allogeneic embryo, a function that is largely attributed to the placental trophoblasts that directly interface with uterine cells and maternal blood.

As with the transcriptomic data, some of the conventional lines had methylation patterns that were more similar to the UCSFB cells than others. A retrospective analysis showed that they were the lines that were derived in physiological hypoxia (Bradley et al., 2010), the strategy we used for the single blastomere derivations. Nevertheless, the lines from the two sources had significantly different methylation patterns at 33% of the loci from the overall comparison (Fig. S3A; Table S4C). They included hypomethylation of UCSFB CpGs in the proximity of *HDAC4* (a histone deacetylase), *HLA-G* (expressed only in trophoblasts; McMaster et al., 1995), other MHC class I genes, *POU5F1*, *SHH*, *SOX1*, *IMPACT*, *PEG3*, *BMP7* and *LIF* (Fig. S3B; Table S4C). In this more restricted analysis, functional enrichments for hypomethylated cytosines in UCSFB5-7 were in gene regions that controlled trophoblast/placental differentiation and regulation of BMP signaling (Fig. S3C; Table S4D). These results suggested that the UCSFB lines had not silenced crucial determinants of embryonic and extra-embryonic development.

Previously, we showed that aberrant genomic imprinting was widespread across a large panel of hESCs and human iPSCs (Nazor et al., 2012). A comparison of the UCSFB and conventional lines showed that the differentially methylated cytosines were associated with *H19*, *MEG3* and *PEG3*, which are most frequently aberrantly imprinted in hESCs and human iPSCs (Fig. S3D, Table S4A). With the exception of aberrant methylation of *PEG3* in one of the nine UCSFB lines, the methylation status of nearly all the known imprinted regions was consistent with the normal hemi-methylated state that is observed in human tissues and primary cell cultures. In addition, a number of imprinted genes were also differentially expressed between the UCSFB and conventional hESCs (Table S3A). For example, *DLX5*, *GLIS3*, *CPA4*, *H19*, *OSBPL5* and *DLK1* were expressed at significantly lower levels in the UCSFB lines, whereas *SNORD108*,* PEG3*, *PEG10*, *MEG3* and *NNAT* were expressed at higher levels (Fig. S3E). For *PEG3*, differences were also observed at the protein level; UCSFB7 immunostained brightly for this molecule compared with the control cell line WA09 (Fig. S3F). Previously, we reported that, among imprinted genes, aberrant methylation of *PEG*3 uniquely associated with aspects of the derivation method (e.g. whether intact embryos were plated and at what stage; Nazor et al., 2012). The results described here provide additional insights into this result in terms of when aberrations in imprinting might arise.

### Sequence determinants of differential methylation

**Fig. 4.**
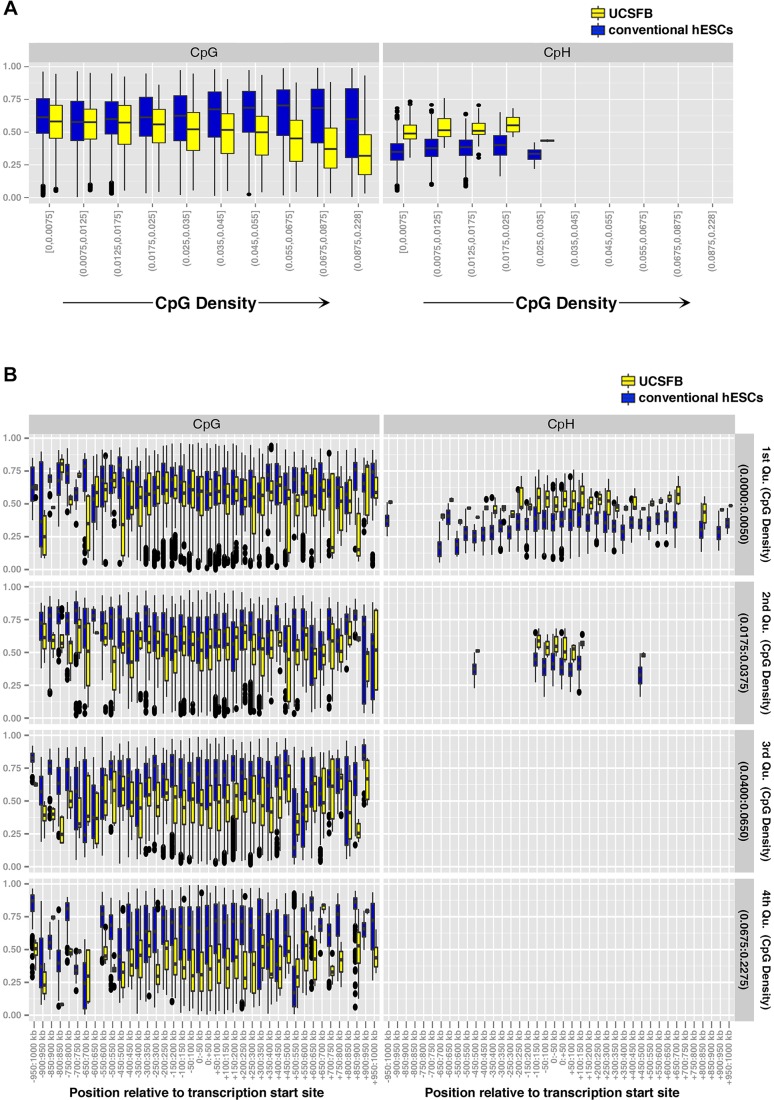
**Sequence determinants of methylation in conventional and blastomere-derived hESCs.** (A) The significance of differential methylation increased as a function of CpG density (left). CpH methylation was significantly lower in conventional hESCs versus lines that were derived in physiological hypoxia and was restricted to regions of low CpG density (right). (B, left) For the UCSFB lines, the hypomethylated CpGs were enriched within a 1 MB window, centered on the transcription start site (TSS)±∼500 kb, in regions of high CpG density (fourth quartile for all 450 K probes) (left graphs). In regions of very low CpG density (first quartile), CpH methylation (right graphs) was spread across the same 1 MB window. In regions of higher CpG density (second quartile), this modification became restricted to the TSS and flanking regions.

CpG density regulates gene expression via DNA methylation (Mikkelsen et al., 2007; Fouse et al., 2008). Plotting the distribution of Beta Values for conventional hESCs and UCSFB5-7, showed that the significance of differential methylation increased as a function of CpG density (Fig. 4A, left). Unlike CpG methylation, which is symmetrical on both DNA strands, CpH methylation is asymmetric and decreases with differentiation (Laurent et al., 2010). CpH methylation was significantly lower in conventional hESCs versus the blastomere-derived lines and was restricted to regions of low CpG density (Fig. 4A, right). For the UCSFB lines, the hypomethylated CpGs were enriched within a 1 MB window, centered on the transcription start site (TSS), in regions of high CpG density (fourth quartile among all 450 K probes; Fig. 4B, left). In regions of very low CpG density (first quartile), CpH methylation was spread across the same 1 MB window. In regions of higher CpG density (second quartile), this modification became restricted to the TSS and flanking regions (Fig. 4B, right). Collectively, these data suggested that differential methylation between UCSFB and conventional hESC lines, which is non-random, might contribute to global differences in DNA architecture among these pluripotent cell types.

### Variations in gene expression among blastomere-derived hESCs

**Fig. 5.**
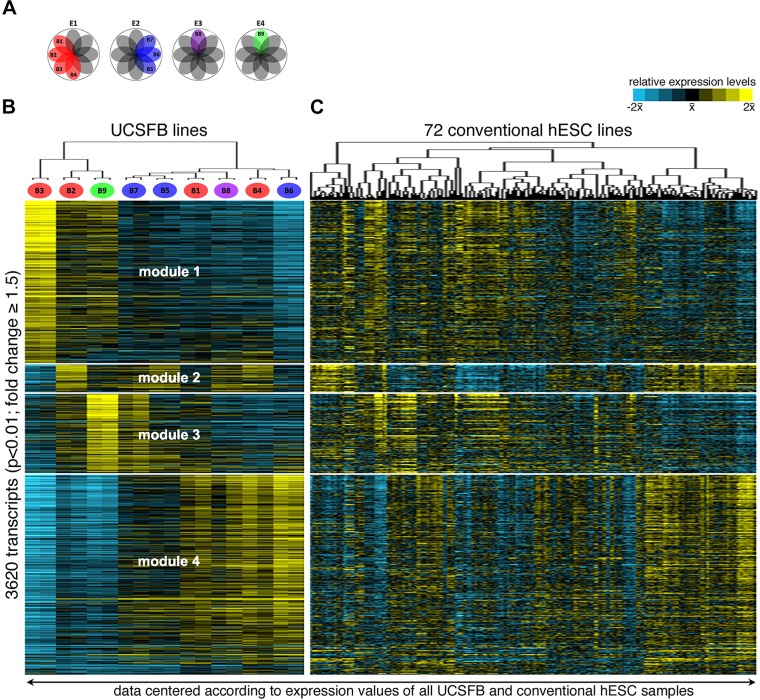
**Gene expression patterns diverged among the UCSFB lines.** (A) The UCSFB lines were derived from embryos that were donated by one couple. The ‘pinwheel’ diagrams depict the single blastomere derivation scheme. In some cases, multiple hESC lines were established from the same embryo (red and blue cells). (B) An ANOVA analysis (*P*<0.01, fold change ≥1.5) comparing the transcriptomes of the UCSFB lines identified 3620 mRNAs as differentially expressed. The heatmap was created by plotting the results from weighted gene correlation network analysis according to rank. Four covariant gene expression modules emerged. As shown in Fig. S4, they included transcripts encoding genes involved in: (module 1) the cell cycle and cholesterol biosynthesis; (module 2) MYC targets and cell type-specific pathways; (module 3) hypoxia responses, glucose metabolism and catabolic responses; (module 4) extra-embryonic development and migration. (C) A heatmap of expression data for the same genes from 72 conventional lines revealed hESCs with portions of these modules, but not the entire program. B, blastomere; E, embryo.

There is growing evidence of blastomere heterogeneity starting as early as the 4-cell stage (Condic, 2014). Thus, we asked whether there were differences among the transcriptomes of the UCSFB lines, which were established at the 8-cell stage (Fig. 5A; color-coded by embryo of origin). To answer this question, we performed an ANOVA analysis, which identified 3620 transcripts that were differentially expressed (FC≥1.5, *P*<0.01; Table S5A). Next, we performed weighted gene correlation network analysis (WGCNA) of these transcripts, which identified four covariant gene clusters (modules 1-4; Fig. 5B; Table S5B). Hierarchical clustering of the UCSFB samples showed that, in some cases, lines that were established from different embryos were more closely related than lines that were derived from the same embryo (Fig. 5B; Table S5A). Subsets of the 72 conventional lines expressed portions of these modules, but not the entire transcriptional program (Fig. 5C).

Analysis of the genes within each module showed that they were involved in many fundamental processes and pathways (Fig. S4, Table S5C). In module 1, we observed differential expression of mRNAs encoding: (1) cell cycle, mitotic and transcriptional regulators; (2) genes involved in cholesterol metabolism; (3) SOX2 targets in hESCs and genes downregulated by CD5. Module 2 was enriched in MYC targets and transcripts of specific cell types, including keratinocytes, fibroblasts and hematopoietic stem cells. Module 3 was unique in the abundance of mRNAs that encoded components of catabolic pathways and glucose metabolism. Module 4 was enriched in transcripts that play a role in migration and amino acid transport. Among the modules, divergence in transforming growth factor beta (TGFβ), platelet-derived growth factor (PDGF) and WNT signaling pathways was apparent. Likewise, modules 3 and 4 were distinguished by transcripts with opposite patterns of E-cadherin (cadherin 1) regulation, increasing or decreasing in expression, respectively. We also noted that certain functions were apportioned across multiple modules. For example, modules 1-3 had mRNAs encoding different elements of hypoxia response pathways, which in some cases, had diametric actions. Modules 1 and 4 were enriched in transcripts that control different aspects of extra-embryonic development. Every module contained mRNAs that have been implicated in specific early differentiation programs that were, for the most part, unique to that module. Thus, the UCSFB lines differed in their expression of transcripts that are thought to play important roles during early development.

For seven of the lines, a subset of the data was confirmed by qRT-PCR, which showed, in all cases, concordance with the microarray results and confirmed differences in mRNA levels among the lines (Fig. S5). For example, the UCSFB lines showed differential expression of crucial lineage-specifying factors including *GDF15* (growth differentiation factor 15), *EOMES*, *FOXA1* (forkhead box A1), *T* (brachyury in mouse), *PDGFB* and *C* (platelet-derived growth factors B and C), *STRC* (stereocilin) and *TIMP4* (TIMP metallopeptidase inhibitor 4; data not shown). Together, these data showed significant differences in gene expression patterns among the lines that could have important consequences in terms of differential responses to physiological hypoxia, cell-cell adhesion and metabolic shifts. We also found evidence of disparate expression patterns of cell cycle regulators and signaling cascades that regulate mouse ESC fate (Habib et al., 2013).

### Differential expression of EOMES, T, GDF15 and active β-catenin among blastomeres of human embryos

**Fig. 6.**
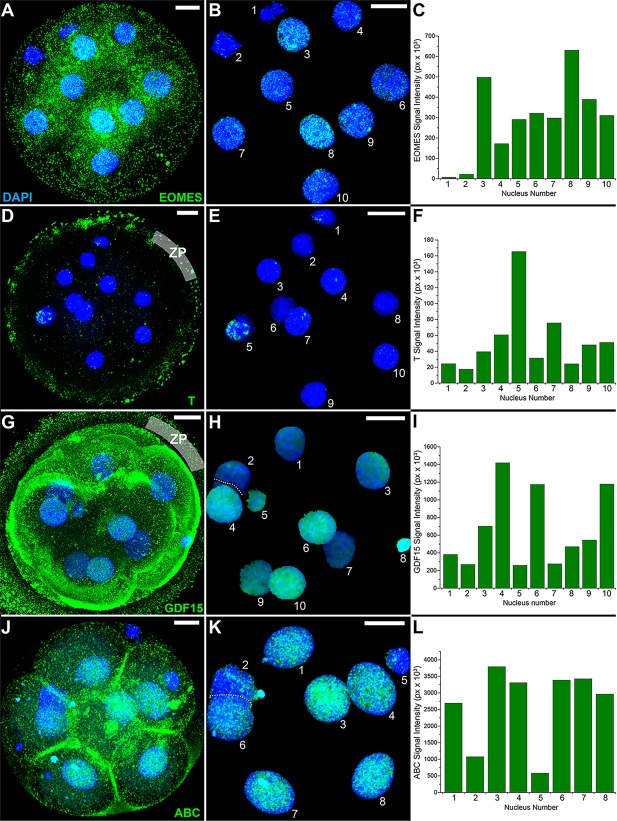
**Immunoanalyses of cleavage-stage human embryos revealed differential nuclear staining for EOMES, T, GDF15 and active β-catenin among blastomeres.** Microarray analyses showed that mRNAs encoding *EOMES*, *T* and *GDF15* were differentially expressed among the UCSFB lines (Fig. S5), suggesting possible differences in blastomere expression of these transcription factors. Additionally, the WNT pathway appeared to be activated in only a portion of the lines (Fig. S4), predicting possible asymmetric distribution of active β-catenin (ABC)*.* An immunolocalization approach was taken to localize these molecules using the antibodies listed in Table S1. The binding of primary antibodies was detected by using species-specific secondary antibodies and nuclei were stained with DAPI. Six embryos were examined for expression of each antigen with the same result. (A,D,G,J) The confocal images of entire embryos were merged into a single micrograph. To image the interior of the embryo shown in G, the surface micrographs at opposite poles were omitted from the merged image. (B,E,H,K) The nuclear staining patterns were extracted from the merged images. (C,F,I,L) Immunostaining was quantified using Volocity software. The dotted lines show division of the nuclei for the purpose of quantification. (A-C) EOMES cytoplasmic and nuclear immunoreactivity was variable (high to low) among the blastomeres. (D-F) Immunostaining for T primarily localized to a single nucleus. Immunoreactivity (presumably nonspecific) was also detected in association with the zona pellucida (ZP). (G-I) Immunolocalization of GDF15 revealed stronger staining in some nuclei and weaker antibody reactivity in others. This growth factor was also detected in the cytoplasm, particularly near the embryo surface, and in association with the ZP. (J-L) As expected, anti-ABC localized to the plasma membrane, but also strongly stained a subset of the blastomere nuclei. px, pixels. Scale bars: 50 µm.

Next, we investigated whether differential gene expression among the UCSFB lines reflected differences in blastomere expression of the corresponding proteins. We immunolocalized EOMES, T, GDF15 and active β-catenin, all of which play important roles in fate specification, in human embryos at the 8- to 10-cell stage (*n*=6/antigen). Confocal microscopy showed some nuclei with relatively high levels of EOMES immunoreactivity; others stained at an intermediate intensity or did not react (Fig. 6A-C; mapped in Fig. S6A). The same range was noted among all the embryos studied with no apparent spatial arrangement. By contrast, staining for T was always strongest in a single blastomere (Fig. 6D-F; mapped in Fig. S6B), a pattern that was common to all the embryos. Likewise, anti-GDF15 (Fig. 6G-I; mapped in Fig. S6C) reacted with only a subset of the blastomere nuclei. Additionally, this growth factor was detected in the cytoplasm with the strongest staining at the periphery. An antibody that recognized active β-catenin (ABC) strongly stained a majority of the nuclei with the others displaying weaker immunoreactivity (Fig. 6J-L; mapped in Fig. S6D). As expected, ABC also localized to the plasma membranes. Together, these results suggested that differences in gene expression among single blastomere-derived hESCs reflected differences in protein expression among blastomeres of early-stage human embryos.

### Derivation and characterization of a human trophoblast stem cell line

**Fig. 7.**
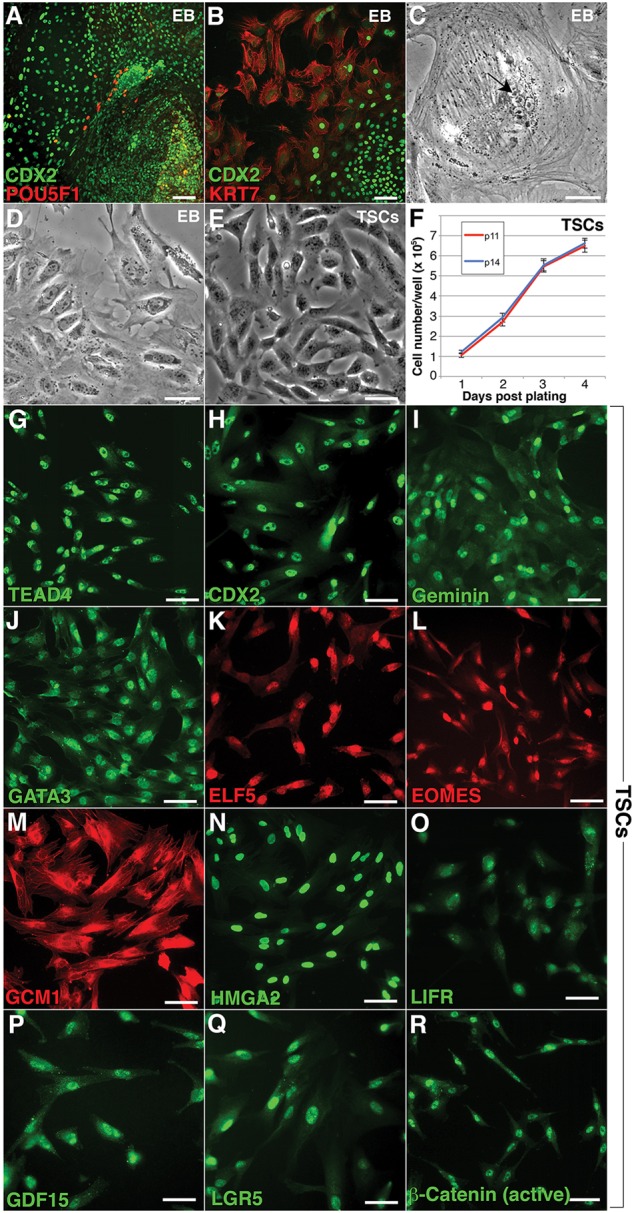
**UCSFB6 EBs spontaneously formed CDX2-positive cells, which gave rise to human trophoblast stem cells.** (A) By day 3, adherent EBs produced large outgrowths in which downregulation of POU5F1 (OCT4) was associated with upregulation of CDX2 expression in a nuclear pattern. (B) Cells at the periphery of outgrowths that no longer expressed CDX2 stained with anti-KRT7, a trophoblast antigen. (C) In some areas of the outgrowths, phase contrast microscopy revealed cells that appeared to have fused (arrow), consistent with a syncytiotrophoblast identity. (D) Other areas of the outgrowths were composed of mononuclear cells with membrane projections indicative of migration, a property of invasive cytotrophoblasts. (E) Areas corresponding to the CDX2-positive outgrowths (shown in A) were manually dissected and propagated under conditions that enabled derivation of human trophoblast progenitors from the chorion (see Materials and Methods). A phase contrast image of the cultures at p11 showed a mononuclear population. (F) Plating of the cells at either p11 or p14 showed that they grew at a consistent rate. (G-I) In an undifferentiated state, they exhibited nuclear staining for transcription factors that drive a trophoblast fate: TEAD4, CDX2 and GEMININ. (J-M) They also immunostained, in a nuclear pattern, for GATA3, ELF5, EOMES and GCM1, transcription factors that are required at later stages of trophoblast differentiation. (N-Q) The cells expressed other stem cell and trophoblast markers including HMGA2, LIFR, GDF15 and LGR5. (R) They also displayed nuclear expression of the active form of β-catenin, which is required for the generation of implantation competent trophoblasts. Scale bars: 100 µm in A,B; 50 µm in C-E,G-R. TSCs, trophoblast stem cells.

Genes controlling extra-embryonic/trophoblast development were differentially expressed/hypomethylated in the UCSFB lines (Figs 2, 3) and *PEG3*, which is highly expressed in TBs, was hemi-methylated as in the placenta (Fig. S3D; Hiby et al., 2001). Therefore, we investigated their TB potential by forming EBs from UCSFB5-7 or control WA09 hESCs. At day 3, immunostaining of UCSFB-derived EBs for POU5F1 showed that expression was limited to a few cells at the center. Towards the periphery, loss of immunoreactivity was accompanied by a striking upregulation of nuclear CDX2 (Fig. 7A). Staining for cytokeratin 7 (KRT7; also known as CK7), a TB antigen, was upregulated in the same areas as CDX2 expression was downregulated, suggesting differentiation of that lineage (Fig. 7B). At days 3 and 5, conditioned medium (CM) from the three UCSFB lines contained higher levels of human chorionic gonadotropin than CM of WA09 cells, with UCSFB6 secreting the highest amount (Fig. S7A). This result was in accord with the qRT-PCR analyses. Compared with the other three lines, UCSFB6 had the highest levels of mRNAs encoding the trophoblast markers CDX2, KRT7 and hCG (human chorionic gonadotrophin β; also known as CGB) (Fig. S7B)*.* Immunoanalyses of UCSFB6 EBs at day 6 showed that the cultures contained cells with the antigenic profile of human TB progenitors (Genbacev et al., 2011). They expressed GCM1 (glial cells missing homolog 1), HMGA2 (high mobility group AT-hook 2), GATA3 (GATA binding protein 3) and GDF15 (growth and differentiation factor 15; Fig. S7C-F). We also observed multinucleated cells, indicative of syncytiotrophoblast (STB) formation (Fig. 7C) and mononuclear cells that migrated away from the EBs, consistent with generation of extravillous/invasive cytotrophoblasts (CTBs; Fig. 7D). UCSFB6 formed the highest number of TB outgrowths, sometimes covering nearly the entire plate; WA09 formed very few. Therefore, we concluded that UCSFB6 robustly formed TBs.

Next, we investigated whether we could derive human trophoblast stem cells (TSCs) from UCSFB6 EBs. At day 3, cells in the outgrowths, corresponding to the CDX2-positive areas shown in Fig. 7A, were manually dissected and cultured using the method we devised for establishing lines of TB progenitors from the human placenta (Genbacev et al., 2011). The morphology of the cells at passage (p) 11 as seen by phase contrast microscopy is shown in Fig. 7E and their growth characteristics at p11 and p14 are graphed in Fig. 7F. With regard to their antigenic phenotype, the cells expressed a wide array of factors that are required for proper placentation in mice (Roberts and Fisher, 2011). In this regard, they immunostained, in a nuclear pattern, for transcription factors that are required for generation of the trophoblast lineage (TEAD4, CDX2 and geminin; Fig. 7G-I) or that are required at later stages of trophoblast differentiation (GATA3, ELF5, EOMES and GCM1; Fig. 7J-M). The nuclei expressed other stem cell and trophoblast markers including HMGA2, LIFR, GDF15 and LGR5 (Fig. 7N-Q). They also displayed nuclear expression of the active form of β-catenin, which is required for the generation of implantation-competent trophoblasts (Fig. 7R; Xie et al., 2008). However, the CDX2-positive cells failed to co-express T (data not shown). When conventional hESCs are treated with BMP4 they form progeny that express both markers, which are thought to be either extra-embryonic mesoderm (Bernardo et al., 2011) or trophoblasts (Roberts et al., 2014). With rigorous manual dissection, the cells were propagated for 20 passages without changing their antigen profile. Thus, we concluded that the phenotype of these cells was consistent with a TSC identity.

**Fig. 8.**
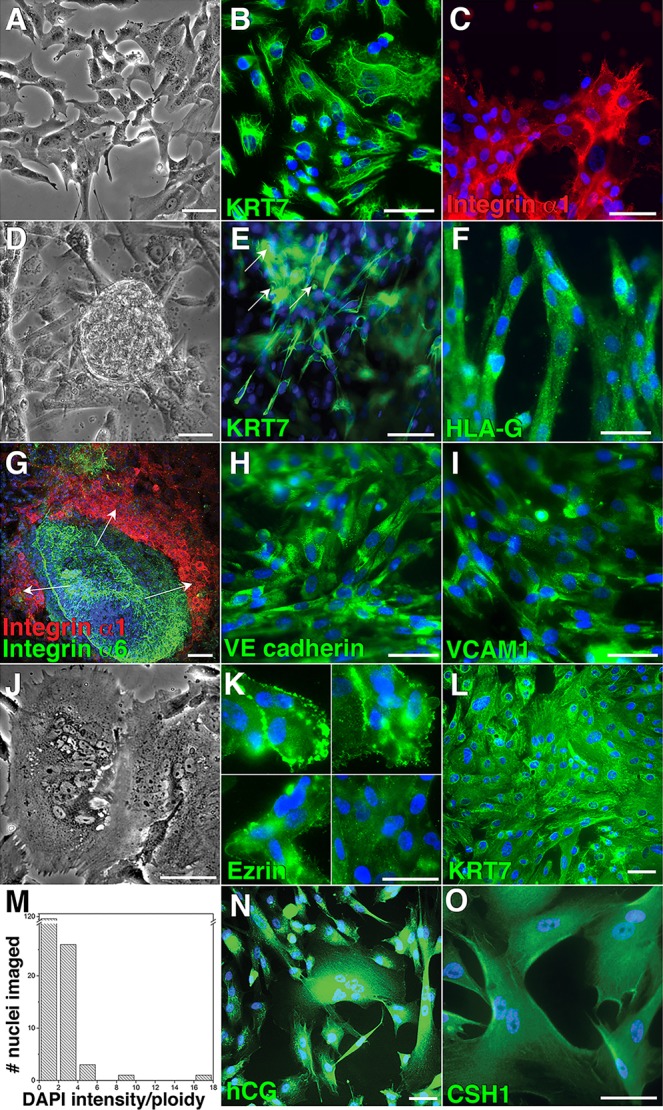
**UCSFB6-derived human trophoblast stem cells formed the mature cell types that carry out the specialized functions of the human placenta.** (A) Upon differentiation, the TSCs formed mononuclear invasive cytotrophoblasts. When they were plated on a thin coating of Matrigel, phase contrast microscopy showed numerous lamellipodia, a feature of migrating cells. (B,C) As the cells moved, they continued to express cytokeratin (KRT7) and upregulated integrin α1, which is required for this process (Zhou et al., 1993). (D) Plating the differentiating TSCs on a Matrigel plug on top of a Transwell filter promoted their aggregation, which was visualized by phase contract microscopy. This behavior mirrors that of primary cytotrophoblasts when they are plated under the same conditions. (E) The aggregates gave rise to invasive cytotrophoblasts that penetrated the Matrigel and migrated through the filter pores to the underside, where numerous KRT7-positive processes were visible (arrows). (F) The invasive cytotrophoblasts immunostained for HLA-G. Among all normal human cells, expression of this MHC class Ib molecule is limited to this trophoblast subpopulation. (G) They also exhibited the integrin α6→α1 transition that accompanies uterine invasion (Zhou et al., 1993). Arrows indicate direction of CTB migration/invasion. (H,I) The cytotrophoblasts executed the unusual epithelial-to-endothelial transition that accompanies invasion (Zhou et al., 1997), exemplified by the upregulated expression of VE cadherin and VCAM1. (J) Upon differentiation, the TSCs also formed multinuclear syncytiotrophoblasts. (K) Fusion was confirmed by immunolocalization of Ezrin, which showed that the nuclei were not separated by plasma membranes. (L,M) DAPI staining was used to estimate the ploidy of the nuclei in KRT7-expressing cells, which showed that a substantial number were hyperdiploid, another unusual feature of human trophoblasts (Weier et al., 2005). (N,O) The TSC progeny also immunostained for hCG and CSH1 (hPL). Scale bars: 50 µm.

Finally, we were interested in their ability to form the mature trophoblast cell types of the human placenta. When the cells were plated on a thin coating of Matrigel, which stimulates CTB migration, phase contrast microscopy showed that they extended processes that were consistent with cell movement (Fig. 8A), which was confirmed by videomicroscopy (data not shown). They continued to express KRT7 (Fig. 8B) and immunostained for integrin α1, which is required for their movement (Fig. 8C; Zhou et al., 1993). We also plated the cells on a thick Matrigel plug atop a Transwell filter, which enables trophoblast invasion. Under these conditions, they formed aggregates (Fig. 8D) that generated invasive cells, which traversed the filter pores to reach the underside (Fig. 8E). The invasive cells upregulated the expression of the MHC class 1b molecule HLA-G (Fig. 7F). Among all normal human cells, expression of this antigen is limited to chorionic and invasive extravillous CTBs as well as amniocytes (McMaster et al., 1995). They also executed the integrin α6→α1 switch that accompanies TB invasion (Fig. 8G; Damsky et al., 1994). Finally, the cells upregulated the expression of VE-cadherin (cadherin 5) (Fig. 8H) and VCAM1 (Fig. 8I) as do invasive CTBs *in situ* and *in vitro*, part of the unique differentiation program in which they execute an epithelial-to-endothelial phenotypic switch (Zhou et al., 1997).

The TSCs also formed multinucleated STBs (Fig. 8J). Fusion was demonstrated by staining the cells with anti-ezrin, which confirmed the absence of plasma membranes between the nuclei (Fig. 8K). We also correlated DAPI staining (Fig. 8L) with ploidy by using a method that was developed for analysis of mouse trophoblast giant cells, which undergo endoreduplication (Gasperowicz et al., 2013). The results showed that a substantial subpopulation of the cells were hyperdiploid (Fig. 8M), another unusual feature of trophoblasts (Weier et al., 2005), which they share with megakaryocytes, cardiomyocytes and hepatocytes (Leslie, 2014). Both mononuclear and multinuclear cells stained for human chorionic gonadotropin and human placental lactogen (CSH1) (Fig. 8N,O). Upon differentiation, the cells failed to express either SOX17 or FOXA2 (data not shown), features of the extra-embryonic mesoderm progeny of BMP4-treated hESCs (Bernardo et al., 2011). Thus, we derived TSCs from UCSFB6, suggesting that this line formed embryonic and extra-embryonic derivatives.

## DISCUSSION

Fundamental questions about preimplantation development remain. In many species, cell fate is tied to spatial orientation: blastomeres on the embryo's outer surface polarize and become trophectoderm, whereas those on the inside assume an ICM fate. Functional dichotomies are evident even earlier (Condic, 2014). Single blastomeres of 2-cell, but not 4-cell, mouse embryos can generate an entire animal. Yet, up to the 8-cell stage, a blastomere can contribute to every cell and organ of the mouse. Thus, fate is restricted long before the blastocyst stage (70-100 cells) when TEAD4 and CDX2 specify TE allocation, and geminin, among other molecules, is required for ICM formation. However, the underlying mechanisms are largely unknown. We reasoned that hESC lines derived from single blastomeres of cleavage-stage embryos might have unique properties that could, in comparison to hESCs derived from later-stage embryos, give us insights into the pathways involved.

Previous reports of hESCs derived from single blastomeres of 4- or 8-cell-stage human embryos described their similarities to conventional lines (Klimanskaya et al., 2006; Chung et al., 2008; Giritharan et al., 2011; Galan et al., 2013). Our study design, which entailed analyses of nine genetically related/identical UCSFB lines, revealed their unique properties. As we had originally hypothesized, the differences were probably due to the fact that each founding cell was removed from an early cleavage-stage embryo, which is in contrast to conventional lines, which are derived by plating intact blastocysts and waiting days to weeks until hESC colonies appear. By contrast, the UCSFB lines were derived from blastomeres that began dividing immediately, rapidly forming colonies without going through formation of an epiblast-like structure, the proposed origin of lines generated from intact blastocysts (O'Leary et al., 2012). Additionally, they were not subject to signals from other cell types that were transiently present in the cultures. Thus, we expected their transcriptomes and methylomes to reflect their unique origins.

Compared with hESCs derived from intact blastocysts, the UCSFB lines had a distinct transcriptome that was enriched in genes involved in trophoblast/ectoplacental cone pathways (Fig. 2C), our first indication that these cells might have trophoblast potential. The blastomere-derived lines were also enriched for components of cholesterol metabolism, possibly underscoring the importance of rapid plasma membrane assembly during early embryo development. Conversely, the conventional lines upregulated the expression of genes that are involved in myriad morphogenic and developmental processes, suggesting the relatively naïve state of the blastomere-derived lines. The finding that conventional hESCs have initiated fate specification aligns with the concept that ESCs derived from blastocysts contain cells in various metastable states, biasing them for differentiation along certain lineages (Graf and Stadtfeld, 2008). This phenomenon has been attributed to fluctuating *Nanog* levels (MacArthur et al., 2012). We hypothesize that the genes that comprise these upregulated pathways offer glimpses into the mechanisms that progressively restrict fate during embryogenesis.

Consistent with the transcriptomic data, the UCSFB lines were relatively hypomethylated in gene regions that are involved in (spongio)trophoblast differentiation and basic cellular processes that are crucial to early development, including cytoplasmic organization, blastoderm segmentation and embryo axis formation (Fig. 3). The hypomethylated CpGs were significantly enriched for associations with genes that are highly expressed in extra-embryonic cell types, including HLA-G and PEG3. Likewise, the gene region near ELF5, which enforces transcriptional networks that define an epigenetically regulated stem cell compartment in the human placenta (Hemberger and Pedersen, 2010), was also hypomethylated (data not shown). Conversely, conventional hESCs were hypermethylated at several loci, including CpGs in the proximity of *HDAC4*, *SHH*, *SOX1*, *POU5F1*, *BMP7*, *IMPACT* and *LIF*.

Comparing the transcriptomes of the UCSFB hESCs showed differences among the lines, even those established from the same embryo. These included variations in the levels of mRNAs that encode fate determinants, such as *FOXA1*, *EOMES*, *T* and *GDF15* (Fig. S5), and in major signaling pathways, including WNT (Fig. S4). We were interested in whether or not these observations were indicative of similar differences in expression of these molecules, at the protein level, among blastomeres of 8- to 10-cell-stage embryos. Immunolocalization of EOMES, T, GDF15 and active β-catenin showed this to be the case. This finding has several implications. EOMES immunolocalizes to all nuclei of preimplantation mouse embryos up to the blastocyst stage (McConnell et al., 2005). Thus, as discussed for POU5F1 in the introduction, this is another example of species-specific differences, between mouse and human, in the expression of fate determinants during the early embryonic period. What do the EOMES, T, GDF15 and active β-catenin immunostaining patterns signify? Although we cannot draw definitive conclusions from our data, these results could indicate that cells expressing these transcription factors have begun to assume fates that are consistent with the pathways they regulate (see below). It may follow that the UCSFB lines that have the highest expression of these factors will be most useful for modeling these lineages. Whether other transcripts that are differentially expressed among the lines are also differentially expressed, at the protein level, among human blastomeres is an interesting question that we are exploring.

Beyond differences in mRNA and protein levels, weighted gene correlation network analysis revealed very interesting findings, for example, in the case of mRNAs encoding gene products that are involved in hypoxia responses. Although *HIF1A* mRNA levels were higher in the UCSFB cells compared with conventional hESCs, they were not significantly different among the blastomere-derived lines. However, HIF1α targets showed substantial variations. Module 1 of the differentially expressed transcripts (Fig. 5; Fig. S4) included genes that are downregulated upon HIF1α overexpression and module 3 included transcripts that are downregulated following HIF1α knockdown. This suggested the possible divergence of HIF1α-mediated O_2_ responses among blastomeres and the lines they generated. Whether or not this observation is related to our previous finding that physiological hypoxia differentially promotes trophoblast expansion remains to be determined (Genbacev et al., 1997). Likewise, genes downstream of *CD5* were under negative regulation in module 1 and positive regulation in module 4. Thus, we have evidence of differences in the lines at transcriptional and pathway levels that may be useful for exploring theories regarding the hierarchy of molecules that regulate early developmental decisions in humans.

Some of the most compelling evidence that the transcriptomic and methylation signatures of the UCSFB lines correlated with differences in their potential came from experiments in which we used directed differentiation protocols to compare the ability of UCSFB5-7 (derived from a single embryo) to form dopaminergic neurons, cardiomyocytes and pancreatic endocrine precursors (Fig. 1A-C). From the results we concluded that the UCSFB lines, as a group, had the same developmental potential as conventional hESCs in terms of forming the embryonic cell types we explored. However, differences in potential among the lines were also apparent. UCSFB7 failed to form dopaminergic neurons *in vitro* (Fig. 1A) and, upon transplantation, endocrine precursors formed from UCSFB6 produced fewer insulin-secreting cells (Fig. 1C). These conclusions were reinforced by the results of parallel experiments in which we studied the unusual ability, compared with conventional hESC lines, of EBs formed from UCSFB5-7 to form trophoblast spontaneously. This is in agreement with a previous report describing derivation of mouse TSC lines from blastomeres of early-stage embryos (Chung et al., 2006). Initial analyses of UCSFB5-7 showed that UCSFB6 secreted the most hCG (Fig. S7A) and expressed the highest levels of mRNAs encoding the trophoblast markers CDX2, KRT7 and hCG (Fig. S7B). This was in keeping with the fact that UCSFB6 highly expressed module 4 transcripts (Fig. 5), which were enriched for extra-embryonic networks (Fig. S4D). Consistent with these results, UCSFB6 was the most efficient in terms of yielding trophoblast stem cell lines. Thus, these experiments provided evidence that the developmental history of a blastomere-derived line, reflected at transcriptomic and methylation levels, influenced its plasticity *in vitro* as shown by differences in the ability to form embryonic and extra-embryonic descendants.

To our knowledge, this is the first report of the derivation of a human TSC line. Although BMP4 may induce conventional hESCs to form trophoblasts, the cells, which are terminally differentiated, cannot be propagated (Xu et al., 2002; Amita et al., 2013). Thus, this new model will enable a detailed exploration of the mechanisms of human TSC self-renewal and the pathways that generate the mature CTBs and STBs that populate the placenta in normal pregnancy. We also think that this model will be very useful for exploring the etiology of pregnancy complications that are associated with aberrations in trophoblast differentiation.

We considered our findings in the context of the major theories of lineage allocation during embryogenesis; namely, stochastic patterning, related to the regulative capacity of the embryo (Wennekamp and Hiiragi, 2012) and pre-patterning (Zernicka-Goetz, 2011). The latter theory predicts that prior to allocation of cells to either the inside or outer surface of the embryo there is heterogeneity in gene expression among the component cells. Supporting evidence has been accumulating from studies of mouse embryos (Piotrowska-Nitsche et al., 2005; Bischoff et al., 2008; Plachta et al., 2011). A recent analysis that used a noninvasive, heritable, multicolor lineage-tracing strategy to study the fate of mouse blastomeres in relation to each other concluded that there is bias towards either ICM or TB differentiation that persists at later stages (Tabansky et al., 2013). We found that the ICMs of late blastocyst-stage human embryos comprised a surprising array of morphologically distinct cells (data not shown). This heterogeneity, which probably reflects transcriptomic and epigenetic differences, could contribute to the observed functional and morphological differences among hESC lines. At earlier stages, immunolocalization of the fate determinants EOMES, T, GDF15 and active β-catenin, which were differentially expressed among the UCSFB lines, showed that only a subset of the nuclei in 8- to 10-cell-stage human embryos stained. These data supported the aforementioned mouse studies and showed that a similar phenomenon is occurring in human embryos. They also suggested that derivation captured, at least in part, an individual blastomere's transcriptome/epigenome. This concept was bolstered by the fact that the differentially expressed molecules included fate determinants that are involved in lineage segregation and/or the early steps in differentiation. However, we cannot rule out stochastic changes, occurring as an individual cell generated a line.

As regenerative medicine therapies enter preclinical and clinical trials, we will learn whether stem cell aberrations at transcriptional, genetic and/or epigenetic levels are important to clinical outcomes. In this regard, the UCSFB lines were significantly hypomethylated compared with conventional hESCs, which was reflected in equally significant differences in the transcriptomes of lines derived by these different methods. Imprinting, including in the *PEG3* region, which is often lost in hESCs and iPSCs, was preserved in 9/10 UCSFB lines (Fig. S3). iPSCs also have epigenetic aberrations, retaining patterns of the cell types from which they were derived (Ohi et al., 2011; Nazor et al., 2012). Thus, in the near term, second generation hESCs with favorable characteristics such as blastomere-derived lines might be a better therapeutic option. However, the UCSFB lines, which were submitted to the National Institutes of Health Human Embryonic Stem Cell Registry some time ago, are still listed as ‘Pending Review’. Thus, future work in the US with these cells and their TB derivatives will encounter obstacles such as obtaining funding from nonfederal sources.

## MATERIALS AND METHODS

### Derivation of UCSFB lines from biopsied blastomeres of cleavage-stage human embryos

Ten hESC lines were derived from biopsied blastomeres of four 8-cell stage embryos and one 12-cell-stage embryo according to published methods (Chung et al., 2008). The University of California San Francisco Committee on Human Research and the Human Gamete, Embryo and Stem Cell Research Committee approved the derivation protocol and written informed consent was obtained from embryo and tissue donors. The details of the method that we used are described in supplementary materials and methods.

### Characterization of the UCSFB lines

Karyotyping was performed by a CLIA-certified Cytogenetics Laboratory (Children's Hospital and Research Center, Oakland, CA, USA) at p11. Methods for assessing markers of pluripotency and for formation and analysis of EBs and teratomas were described previously (Genbacev et al., 2005). The antibodies, dilutions and sources are summarized in Table S1.

### Generation of neural stem cells and precursors from UCSFB5-7

To derive neural stem cells (NSCs), colonies were harvested using a scraper with care taken to avoid excessive colony fragmentation. The cells were cultured in suspension as EBs for 8 days in standard hESC medium without bFGF. Then the EBs were cultured for an additional 2-3 days in suspension in neural induction medium containing DMEM/F12 with Glutamax (Invitrogen), 1× NEAA, 1× N2 (Invitrogen) and bFGF (20 ng/ml) prior to plating. After 2-3 days, numerous neural rosettes formed. To obtain a pure population of NSCs, the rosettes were manually isolated, dissociated into single cells using Accutase (Invitrogen) and re-plated onto culture dishes. Dopaminergic differentiation was obtained by culturing NSCs in medium conditioned by PA6 cells for 5 weeks (Swistowska et al., 2010) or in xeno-free defined medium for 4-5 weeks on culture dishes or glass coverslips coated with 20 mg/ml poly-L-ornithine (Sigma) and laminin (10 mg/ml) (Swistowski et al., 2009). Expression of SOX1 was evaluated in dissociated neural rosettes by immunolocalization. At the end of the experiment, the cells were stained with anti-β-tubulin isotype III, anti-nestin or anti-tyrosine hydroxylase (see Table S1).

### Generation of cardiomyocytes from UCSFB6

Rotary orbital suspension was used to form 300 EBs of uniform size from single cell suspensions of UCSFB6 and WA09 hESCs (Sargent et al., 2010). Over 20 days, the percentage of adherent EBs demonstrating spontaneous contractile activity in differentiation medium was scored and cardiomyocyte subtypes profiled using a previously described panel of qRT-PCR assays developed for this purpose (Ritner et al., 2011). The experiment was performed twice.

### Generation of pancreatic endoderm cells from UCSFB5-7

Cultures were maintained in mTeSR with 25 ng/ml bFGF in 8% O_2_. *In vitro* differentiation followed the method of Kroon et al. (2008). The upregulation of the expression of SOX17 on day 3 and PDX1 on day 9 was assessed using an immunolocalization approach. The cells were fixed in 4% paraformaldehyde (PFA), permeabilized with 0.1% NP40/0.1% Triton X-100, and nonspecific binding was blocked by incubation with 4% bovine serum albumin in PBS. The appropriate species-specific, Alexa Fluor-conjugated secondary antibodies were from Invitrogen. At day 14, the cells were harvested and 0.5-50×10^6^ were transplanted under the kidney capsules of non-diabetic SCID-beige mice, at least two recipients/line. Sham-operated animals served as controls. Kidneys were recovered after 100 days. To immunostain transplanted cells, grafted kidneys were fixed overnight in 4% PFA at 4°C, then embedded in paraffin and sectioned. They were stained with anti-PDX1 and anti-INSULIN (see Table S1).

### Microarray analyses

hESCs were grown in feeder-free conditions for 11-16 passages. Total RNA was purified from snap-frozen sample pellets using a mirVana miRNA Isolation Kit (Ambion), quantified using the Ribogreen reagent (Lifetech), and then quality assessed on a Bioanalyzer 2100 platform (Agilent). Two hundred nanograms of total RNA was amplified and labeled using the TotalPrep Kit (Ambion). Then the labeled product was hybridized to Illumina HT12v3 beadchips and scanned on a BeadArray Reader (Illumina) according to the manufacturer's instructions. In GenomeStudio, probes were filtered for those detected at *P*<0.01 in at least one sample and exported for normalization in R via robust spline normalization. Differential expression and ANOVA analyses were executed using Limma (Smyth, 2004). Hierarchical clustering was performed using Cluster, with Euclidian distance and complete linkage. Functional enrichments were executed in GREAT (McLean et al., 2010) with the ‘Association rule settings’ set to ‘Single nearest gene’. Microarray data have been deposited in Gene Expression Omnibus under accession number GSE63592.

### qRT-PCR analyses

RNA samples were isolated using an RNeasy Plus Kit (Qiagen). RNA concentration/quality was assessed using a Nanodrop spectrophotometer (Thermo-Scientific). cDNA libraries were prepared with 1 µg of RNA using an iScript kit (Biorad) and diluted 20-fold in water. Taqman qRT-PCR reactions were carried out in triplicate. The primer sequences are listed in Table S2. Reaction specificity was confirmed by determining the melting curve of the products or by gel electrophoresis. Differences among target expression levels were estimated by the ΔΔCT method with normalization to *GAPDH*.

### DNA methylation profiling

DNA was extracted from 1×10^6^ cells (QIAGEN DNeasy Blood and Tissue Kit), quantified (Qubit dsDNA BR Assay Kits, Life Technologies), quality controlled (DNA1000 Kit and BioAnalyzer 2100, Agilent), and bisulfite converted (EZ DNA Methylation Kit, Zymo Research) according to each manufacturer's protocol. Bisulfite-converted DNA was hybridized to Infinium-450 BeadChips (Illumina) and scanned with an iScan (Illumina). Quality control was performed in GenomeStudio. HumanMethylation450 data were normalized using SWAN (Maksimovic et al., 2012) and differential methylation was identified using Limma (Smyth, 2004). Hierarchical clustering was performed using Cluster, with Euclidian distance and complete linkage. Functional enrichments were executed in GREAT (McLean et al., 2010) using default settings. DNA methylation data have been deposited in Gene Expression Omnibus under accession number GSE63592.

### Embryo immunostaining

Six embryos per marker were grown as described above for hESC derivation. They were fixed for 10 min in 4% PFA, washed in PBS and permeabilized by incubation for 20 min in 0.5% Triton X-100 in PBS at room temperature. Nonspecific immunoreactivity was blocked by incubation in 1× TBS/fish gelatin (SurModics)/0.1% Triton X-100 overnight at 4°C. Immunostaining was performed in blocking buffer with anti-T, anti-EOMES, anti-GDF15 or anti-active β-catenin (see Table S1) at 4°C overnight. For evaluating T expression, conventional hESC-derived teratomas and cultured hESCs were used as positive and negative controls, respectively. For evaluating EOMES and GDF15 expression, human trophoblast progenitor cells and conventional hESCs were used as positive and negative controls, respectively. For immunostaining with an antibody that recognized active β-catenin, human placenta was used as a control; invasive cytotrophoblasts are immunoreactive and fibroblasts are not. After washing (2×30 min) in blocking buffer, the embryos were stained with species-specific Alexa Fluor-conjugated secondary antibodies (Invitrogen) in 1 mg Hoechst 33342/ml blocking buffer for 1 h at room temperature. Confocal images were acquired on a Leica TCS SB5 microscope. Individual blastomere nuclei were virtually isolated and the green channels quantified (Volocity 6, PerkinElmer).

### TB differentiation and TSC derivation

Differentiation was via EB formation. Manually dissected cell clumps (UCSFB5-7, WA09) were cultured in Ultra Low Cluster Plates (Costar, Corning Incorporated) in KSR medium with 10% fetal calf serum (FCS) for 6 days. The EBs were plated on 3% Matrigel (BD Biosciences) in KSR medium with 10% FCS. EB outgrowths were cultured for 6 days. TB differentiation was analyzed as described (Genbacev et al., 2011). The antibodies, sources and working concentrations, are summarized in Table S1.

TSC derivation utilized the strategy for establishing human TB progenitors from the chorion (Genbacev et al., 2011). Cells in EB outgrowths formed from UCSFB6 were manually dissected, transferred to gelatin (Sigma)-coated wells and cultured in DMEM/F12 medium supplemented with 10 ng/ml bFGF, 10% FCS and 10 µM SB431542 (inhibitor of activin/nodal signaling; Tocris Biosciences) until they formed tightly packed colonies of CDX2-positive cells as determined by immunostaining. TSC colonies were passaged manually. TSC differentiation to CTBs or STBs and phenotyping of the SCs and their progeny was performed as described (Genbacev et al., 2011).
